# Transcriptional Stages of Conidia Germination and Associated Genes in *Aspergillus flavus*: An Essential Role for Redox Genes

**DOI:** 10.3390/toxins14080560

**Published:** 2022-08-18

**Authors:** Chong Li, Sifan Jia, Shahid Ali Rajput, Desheng Qi, Shuai Wang

**Affiliations:** 1 Department of Animal Nutrition and Feed Science, Huazhong Agricultural University, Wuhan 430070, China; 2 Department of Animal Feed and Production, Faculty of Veterinary and Animal Sciences, Muhammad Nawaz Shareef University of Agriculture, Multan 66000, Pakistan

**Keywords:** *Aspergillus flavus*, conidia, germination, transcriptome, redox genes

## Abstract

Aflatoxin is a threatening mycotoxin primarily present in the agricultural environment, especially in food and feedstuff, and poses significant global health risks. Aflatoxins are produced mainly by *Aspergillus flavus*. Conidia germination is the first step for *A. flavus* development. In this study, the transcriptome of *A. flavus* conidia was analyzed at three different stages of conidia germination, which were characterized by two different microscopes. Dormant conidia grew isotropically with the cell size increasing up to 5 h of after being inoculated in a liquid medium. Conidia changed towards polarized growth from 5 to 10 h of germination, during which germ tubes formed. Moreover, transcriptome analyses revealed that a larger number of genes changed in the isotropic growth stages compared to polarized growth, with 1910 differentially expressed genes (DEGs) up-regulated and 969 DEGs down-regulated in isotropic growth. GO and KEGG pathway analyses and pathway enrichment demonstrated that, in the isotropic growth stage, the top three pathways were translation, amino acid and carbohydrate metabolism. The ribosome was a key pathway in translation, as *RPS28e*, *RPL53* and *RPL36e* were the top three DEGs. For polarized growth stage, lipid metabolism, amino acid metabolism and carbohydrate metabolism were the top three most active pathways. *POX1* from alpha-linolenic acid metabolism was a DEG in lipid metabolism as well. Genes related to the antioxidant system were crucial for conidia germination. Furthermore, RT-PCR results showed the same trends as the transcriptome for redox genes, and essential oils have a significant inhibitory effect on germination rate and redox gene expression. Therefore, redox genes play an important role during germination, and the disruption of redox genes is involved in the mechanism of action of coumalic acid and geraniol against *A. flavus* spore germination.

## 1. Introduction

The mycotoxin contamination of food and agricultural products is a significant threat towards human and animal health and causes enormous economic losses [1]. In particular, aflatoxins are common and one of the most toxic substances in the world [2]. Aflatoxins, including AFB_1_, AFB_2_, AFG_1_ and AFG_2_, are characterized as class A carcinogens by the International Agency for Research on Cancer [3]. *Aspergillus flavus* is one of the primary fungi that produces aflatoxins [4].

Mature *Aspergillus.* spp. produce billions of single-celled dormant conidia that are found all over the world, including in the desert, polar regions or other severe environmental conditions that are not suitable for living [5]. *A. flavus* is not only associated with food and feed spoilage but also acts as an opportunistic pathogen in plants and animals [6,7,8]. Conidia are the main vehicles of distribution for *A. flavus* and are reproductive structures; they are characterized by a dormant state that is essential for survival in hostile conditions [9,10]. Air-dispersed conidia are highly resistant to extreme environments and can remain viable for several years and begin to germinate as soon as they are in hospitable environmental conditions and in the presence of nutrients such as fermentable sugars, inorganic salts and a nitrogen source [11,12]. Therefore, understanding the process of *A. flavus* conidia germination is important for food and feed safety.

The germination of a fungal spore is also an important way for target organisms to be infected during the spoilage of food and feed. Dormant conidia have irregular spherical shapes. Upon the activation of germination, water uptake leads to an increase in intracellular osmotic pressure [13]. For *Aspergillus niger*, during this stage, the first morphological change in conidia germination involves swelling, with the diameter of the spore increasing two-fold or more. The swelling phase of conidia is also called isotropic growth [14]. Swelling is concomitant with many metabolic activities such as respiratory metabolism, amino acid biosynthesis, protein biosynthesis, and so on [15]. Swollen conidia are followed by polarized growth that leads to germ-tube formation. During this phase, the formation of a germ tube is also called polarized growth. A large number of metabolism activities are the same as those found in isotropic growth, and only some special metabolic activities, such as cytoskeleton formation, the vesicle trafficking system and landmark protein, are different [16]. Next, conidia complete germination when the length of the germ tube is equal to the half of the diameter of the spore. At later stages of development, the germ tube grows faster and faster and branching leads to agglomeration, mutually resulting in fungus hypha accumulation. During this phase, the secondary metabolite aflatoxins are major secreted from hypha [17].

RNA-Seq technology has been widely used in microbiology research for investigating the dynamic changes in RNA expression, including conidia germination, mycotoxin biosynthesis, environmental stress response, nutrient metabolism and so on [5,18,19,20]. In this study, the germination rate of *A. flavus* conidia at different times was analyzed to determine the various stages of conidia germination. Subsequently, we used different microscopes to study the morphological changes of *A. flavus* conidia during germination on Czapek–Dox (CZ) culture medium. Then, RNA-Seq technology was used to identify transcriptomic changes in developing conidia involved in various *A. flavus*, and the molecular functions of differentially expressed genes (DEGs) and their metabolic pathways were analyzed using bioinformatic methods. Most changes in the transcriptome occurred during the early phase of germination. The data showed that the transcriptome of the dormant spore is very different from that of conidia during all germination phases. Our study focused on the different changes on translation, carbohydrate and lipid metabolism.

## 2. Results

### 2.1. Conidia Germination Rate

**Figure 1:**
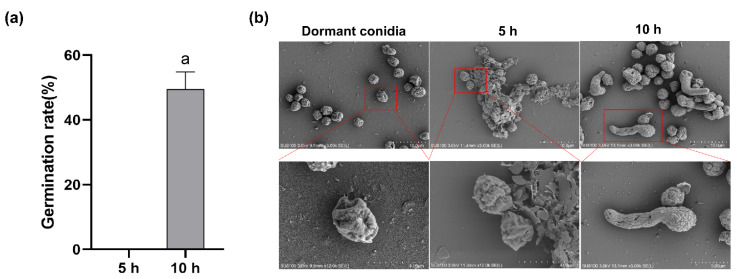
(**a**) Conidia germination rate of *A. flavus* in CZ culture medium. (**b**) Germination of *A. flavus* conidia as observed by SEM. Microscope images are shown for dormant conidia (0 h) and germinating conidia at 5 h and 10 h, respectively. Bar represents 10 µm, 4 µm and 5 µm (10 h). ^a^ Columns with different lowercase letters indicated significant differences between the compared groups (*p* < 0.05).

Conidia germination of *A. flavus* has a maximal rate between 28 and 30 °C. In this study, *A. flavus* spores were inoculated in CZ culture medium at 30 °C, with approximately 50% conidia germination after 10 h (Figure 1a). Isotropic growth (swelling phase) was observed before 5 h after inoculation, and polarized growth (germ tube forming) occurred at 5 h and 10 h (Figure 1b). The cell size of dormant conidia was about 3 × 4 µm, but in the isotropic growth stage, the cell size was much larger than dormant conidia. The morphology of the swelling conidia was different with the dormant conidia, with wrinkle recoveries and flat cell walls. In the polarized growth stage, conidia completed germination when the length of the germ tube was equal to the conidia’ radius, and some conidia had more than one germ tube.

### 2.2. Flow Cytometry

**Figure 2:**
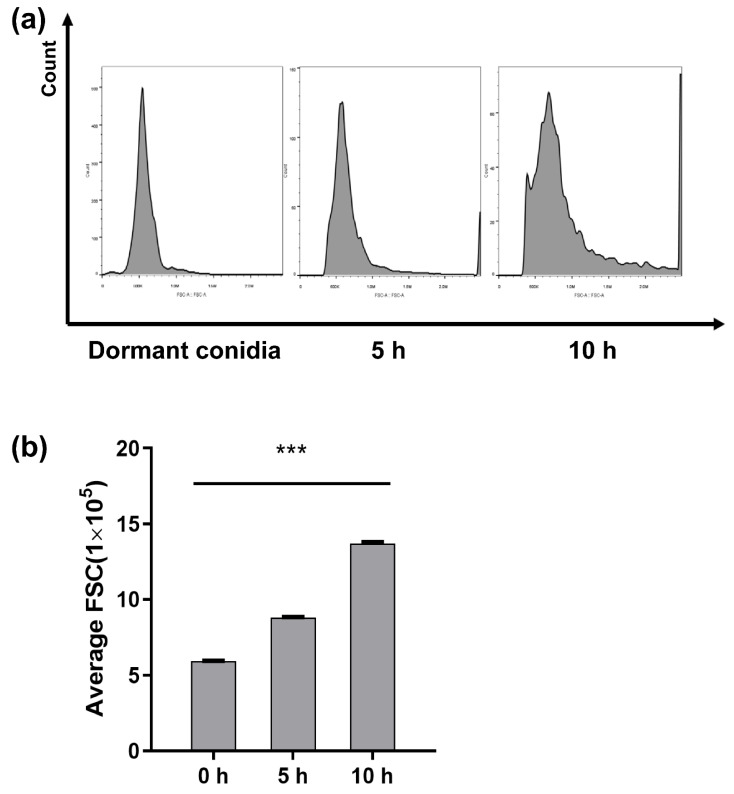
The differences in size of conidia germination under three different stages. The *x*-axis indicates forward scatter (FCS), and the y-axis indicates counts of profiles of 10,000 conidia at 0 h, 5 h and 10 h (**a**). Average size of 10,000 conidia measured as the FSC parameter (**b**). The means and standard errors of duplicate samples have been plotted (*n* = 3). The“*”on column diagram indicate a statistically difference of treatment at “***”means *p* < 0.001.

Conidial samples were prepared and analyzed by flow cytometry over a 10 h period to measure the increase in the size of dormant conidia harvested with PDA. The Flowjo software provided numerical values for the FSCs of the conidial and generated a graph (Figure 2). The counts of conidia demonstrated that evident isotropic growth expansion occurs over the first few hours of germination, and polarity formation and germ tube emergence were also apparent between 5 and 10 h.

### 2.3. Transcriptional Profiling

In this study, nine samples of *A. flavus* NRRL 3357 were sequenced using RNA-Seq technology, averaging 24,136,399 raw sequencing reads and 24,125,185 clean reads after filtering out low quality reads. Table 1 briefly summarizes the information of sequencing data for each sample.

### 2.4. Gene Expression

**Figure 3:**
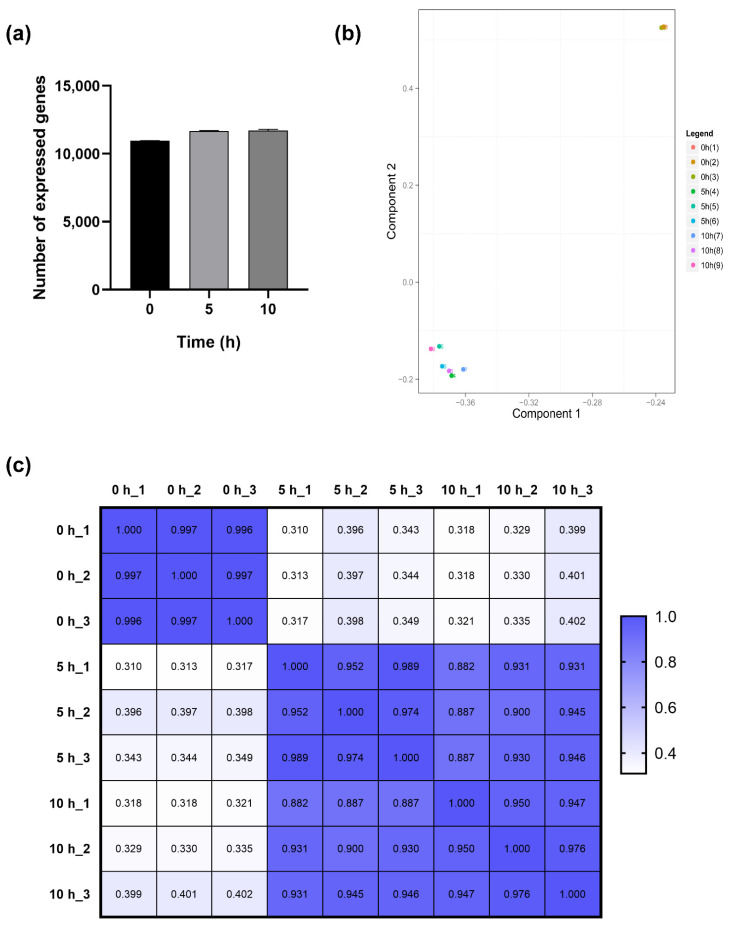
The number of expressed genes (**a**) during germination of *A. flavus* and the similarity of the RNA profiles of the different stages of germination represented by principal component analysis (**b**) and correlation coefficients (**c**).

Gene expression levels were quantified by a software package called RSEM. The number of identified expressed genes was counted and calculated in proportion to the total gene number in the database for each sample in Figure 3a. Dormant conidia averaged 10,966 transcripts. The number of expressed genes increased to 11,656 5 h after inoculation and then gradually increased to 11,702 10 h after germination. According to principal component analysis (PCA) analysis (Figure 3b), the dormant conidia sample differed from all other time points in that it contributes to the majority of the first principal component while the variation in the other time points was predominantly confined to the second principal component. The correlation of expressions (Figure 3c) showed that the RNA profile of dormant conidia was the most different when compared to other samples.

### 2.5. Differential Gene Expression and Functional Analysis

**Figure 4:**
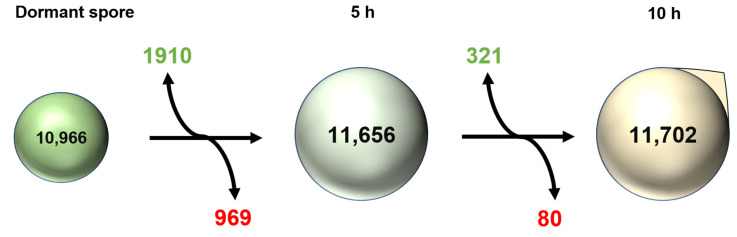
Overview of the global changes in the transcriptome of conidia during germination. Inside the spore, the number of expressed transcripts is provided. Green and red represent numbers of genes with fold change ≥2 up-regulated and down-regulated between two stages, respectively.

The results of the differential gene expression analysis revealed that were many germination responsive genes existing in the spore (Figure 4). Compared to dormant conidia, 1910 genes were up-expressed and 969 genes were down-expressed with a two-fold change or greater (*p* < 0.05). Meanwhile, a number of differentially expressed genes were much lower between the 5 h and 10 h time points. Genes numbering 321 were up-regulated between 5 h and 10 h, whereas 80 genes were down-regulated.

**Figure 5:**
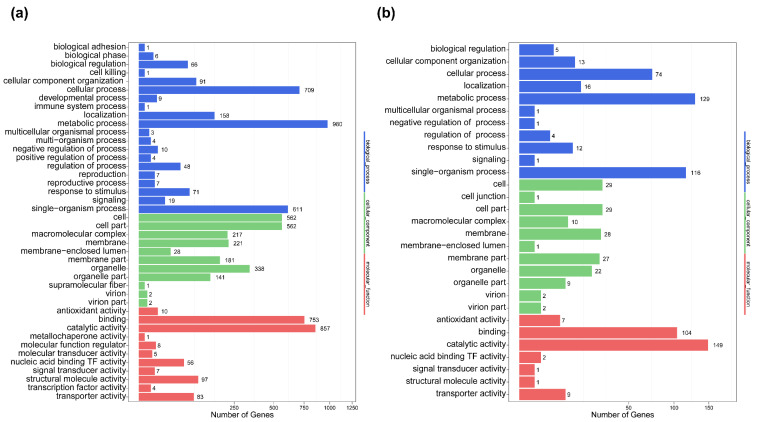
GO functional enrichment analysis of DEGs in 0 h vs. 5 h (**a**) and 5 h vs. 10 h (**b**) during conidia germination. All GO terms are grouped into three ontologies: blue is for biological processes, green is for cellular components and red is for molecular function. The y-axis indicates the subcategories, and the x-axis indicates the number of genes in the same category.

Moreover, compared to dormant and 5 h conidia, GO analysis results indicated that 726 DEGs, accounting for 27.13% of all significant DEGs, were associated with cellular compounds; 1297 DEGs, accounting for 22.32% of all significant DEGs, were annotated with molecular functions; 1207 DEGs, accounting for 22.96%, were classified with biological processes. The groups in the three main categories are shown in Figure 5a. Within the biological process category, the most highly represented groups were metabolic processes, cellular processes, single-organism processes and localization. In the cellular component, cells, cell parts, organelles, membranes and macromolecular complexes were the most abundant groups. Meanwhile, binding, catalytic, structural molecular activity, transport activity and nucleic-acid-binding transcription factor activity were the largest terms with respect to molecular functions. Likewise, the results of GO analysis revealed that metabolic processes, single-organism processes and cellular processes are the most abundant terms between the 5 h and 10 h time point (Figure 5b). Cells, cell parts, membranes, membrane parts and organelles in the cellular component and binding and catalytic activity in molecular functions were the most highly represented terms.

Genes usually interact with each other to play roles in certain biological functions. Pathway enrichment analysis of DEGs based on the KEGG database was performed. After comparisons of dormant with 5 h time point conidia, 1849 genes were annotated for 121 known metabolic and signal pathways. During the late stages of germination (5 h vs. 10 h), 238 genes were classified into 92 pathways. However, the pathway distributions of these changes in genes in both isotropic growth and polarized growth were in accordance with each other, and more genes displayed at least a 2-fold change in isotropic growth. Carbohydrate metabolism, amino acid metabolism, translation, lipid metabolism and metabolism of cofactors and vitamins involved in metabolism and genetic information processing were the most abundant groups.

**Figure 6:**
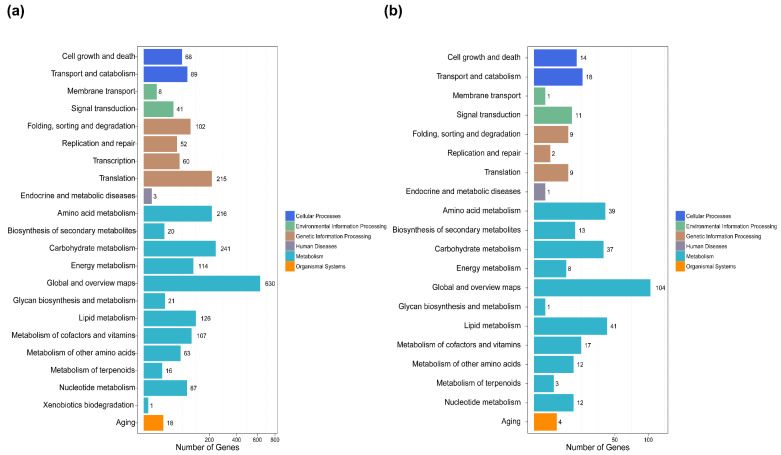
KEGG classification on DEGs for each comparison at 0 h vs. 5 h (**a**) and 5 h vs. 10 h (**b**). X-axis means the number of DEGs. Y axis represents second KEGG pathway terms. All second pathway terms are grouped in top pathway terms indicated with different colors.

**Figure 7:**
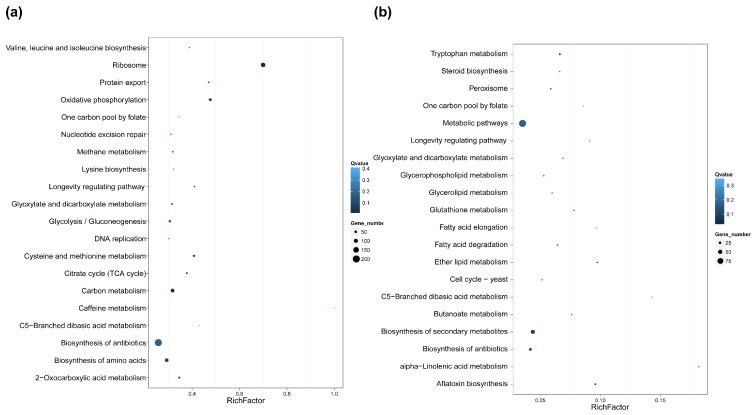
Statistics of pathway enrichment of DEGs in each comparison at 0 h vs. 5 h (**a**) and 5 h vs. 10 h (**b**). Rich factor is the ratio of differentially expressed gene numbers annotated in this pathway term to all gene numbers annotated in this pathway term. A greater rich factor means greater intensiveness. Q-value is corrected *p*-value ranging from 0 to 1, and a lower Q-value means greater intensiveness. We only displayed the top 20 enriched pathway terms.

In addition, the top 20 KEGG enrichment results (shown in Figure 6a) were generated. In the isotropic growth stage (Figure 6b), ribosome- and oxidative phosphorylation-related DEGs were the most significant, which indicated that the initiation of energy metabolism and translation constitute key processes in the initial stages of germination (Figure 7a). Furthermore, the map of ribosome and the most changeable genes are represented in Figure S1; RPS28e was the most up-regulated gene and RPl36e was the most down-regulated gene. During polarized growth stages (Figure 6b), organic acid metabolism and lipid metabolism were the most abundant pathways. These data showed that lipid metabolism was an important process for germ-tube growth (Figure 7b). Furthermore, alpha-linolenic acid metabolism from lipid metabolism was the most important from pathway enrichment analysis. All five DEGs in this pathway were down-regulated and they are shown in Table 2 and Figure S2.

Additionally, the aflatoxin biosynthesis pathway activated in the stage of germination (Table 2 and Figure S3), which means that the secondary metabolism was triggered with the germination process and became ready for aflatoxin biosynthesis.

### 2.6. Antioxidant System during Conidia Germination

**Figure 8:**
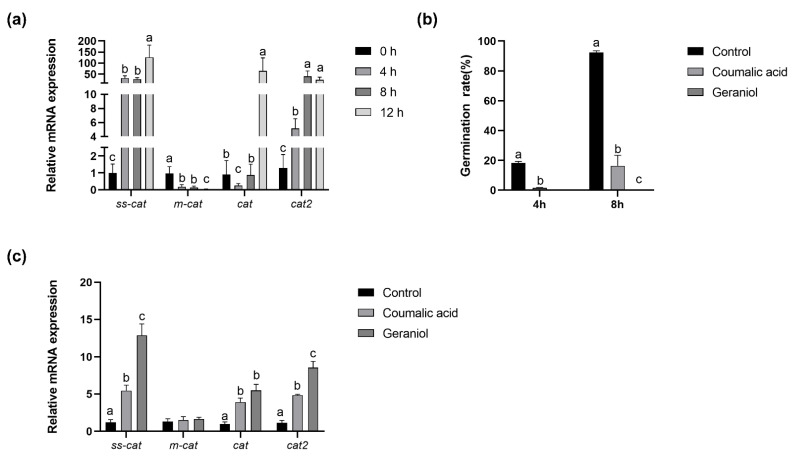
Relative mRNA abundance of redox genes during conidia germination of *A. flavus* (**a**). Germination rate with coumalic acid and geraniol supplement (**b**) during the germination of *A. flavus*. Effect on coumalic acid and geraniol supplement on relative mRNA abundance of redox genes after 8 h germination of *A. flavus* (**c**). Values are mean ± SEM, *n* = 6. Means without a common letter differ, *p* < 0.05.

The identification of the redox gene effect during conidia germination is of paramount importance. The essential oil has been reported to efficiently kill conidia of *A. flavus* via triggering reactive oxygen species and causing redox-balance damage. According to the results of RNA-Seq, the redox gene expression was determined by RT-PCR (Figure 8a) more specifically during conidia germination and separated into four different stages. Real-time qPCR results showed that redox gene mRNA levels of ss-cat and cat2 increased, while m-cat decreased as conidia germination progresses, which was also demonstrated in RNA-Seq results. With the coumalic acid and geraniol supplementation, conidia germination was inhibited as Figure 8b shows, and the mRNA abundance of ss-cat, cat, and cat2 increased.

## 3. Discussion

For the conidia of *A. flavus*, germination is the first crucial step from asexual propagule to vegetative mycelium growth and the production of aflatoxin, which causes contamination and the spoilage of food and feed. Therefore, an improved understanding of the conidia germination process, metabolism and key genes and pathways can provide significant contributions to studies focused on controlling aflatoxin contamination and improving food and feed safety.

The transitions of conidia germination are recognized in three different stages: dormant conidia, isotropic growth and polarized growth [21]. The generated hyphae are then separated into compartments by septa [22]. Each stage has its own unique morphological characteristics. A previous study found that dormant conidia are highly stress-tolerant structures [23], and they are able to survive and germinate under high-pressure conditions such as dehydration, extreme temperature, osmotic pressure variations in pH and UV due to the three layers of the cell wall and several inner characteristics [11,24]. Dormant conidia germinated when flexible nutrients such as sugars, inorganic salt and nitrogen source were supplemented in most *Aspergillus* strains. However, germination times depend on the different culture conditions and variations in different *Aspergillus* strains.

In our study, we chose 5 h after inoculation as the stage of isotropic growth and 10 h germination as the stage of polarized growth, respectively, using a series of microscopes and flow cytometry. While the morphology change was similar to other typical *Aspergillus* such as *A. niger* and *A. fumigatus*, during the isotropic stage, the cell’s size was up to twice that of dormant conidia and the germ tube grew out from one side [25]. When the length of the tube was equal to the conidia’s radius, this meant that conidia germination was successful. The morphological impact of germinating conidia on the surface ultrastructure of *A. flavus* spores was investigated by scanning electron microscope (SEM).

The goal to understand the transcriptome landscape of dormant and geminating conidia of the filamentous fungi *A. flavus* was achieved in this study. Presumably, our research is the first report to analyze the transcriptome levels of the dormant and germinating conidia within the aflatoxigenic *A. flavus* strain. In the current study, the RNA-Seq produced an average of 1.21 billion bp raw data size and 24.1 million raw reads for each treatment, and approximately 10,000 genes were characterized after filtering out low quality reads. The data indicated that the RNA expression level of dormant conidia is substantially different when compared to other stages of germination, each of which is characterized by a typical morphology. The transcriptome of conidia changed gradually before the stage of isotropic growth (swelling), in which the gene’s expression had many variations. The correlation of the expression of the dormant and germinating conidia 5 h after inoculation (0.350) and 10 h after inoculation (0.348), as well as the correlation between the 5 h and 10 h time points, is 0.92, which provides evidence for these changes.

About 23,320 genes were expressed in vegetative growth in a control group of *A. flavus* in different water activity treatments, while transcripts of 33% of the genes were active in dormant conidia of *A. niger* [18] and a similar trend was found in *Aspergillus. fumigatus* [10]. Compared to vegetative hyphae and aerial structures, the complexity of conidial RNA is lower because these spores represent a single cell type [26]. In contrast, mycelium, vegetative hyphae and aerial structures are composed of different types of hyphae and cells. Previous studies have shown that the RNA profile has a few changes after one-year storage in the dormant conidia of *A. fumigatus,* and it was thought that mRNA was in a pre-packed pool stage for the translation and quick response of conidia germination [10]. For instance, a few compounds such as heat shock proteins, trehalose, mannitol and dehydrins in dormant conidia are key for maintaining the structures for surviving extreme conditions [11]. The transcripts of genes for encoding these related proteins were not only highly accumulated but the transcripts of genes related to the synthesis and degradation of compatible solutes were also unique in dormant spores. Similar research has shown that genes involved in the defense of the conidia cell wall (for example, the genes responsible for making hydrophobins and pigmentation [27,28]) are specific for dormant conidia in *A. niger* and *A. fumigatus*. Furthermore, some transcription-factor-related genes that are essential for spore formation and maturation were only found in dormant conidia but absent in germinating conidia [18].

In this study, to evaluate the changes between the breaking of dormancy and dormant conidia in *A. flavus*, RNA-Seq was performed. Compared to other research, significant transcriptional changes occurred over the first 2 h of germination in *A. niger* by using genome-wide microarrays, but the total gene number was only about 4000, which is far less than the 11,000 genes in our study [15]. Other research also revealed that the most significant changes occurred over the initial stages of conidia germination when compared to the subsequent stages of germination in *A. fumigatus* [9]. As a result of this observation, RNA-Seq technology was used to study this period of the breaking of dormancy in more detail and as a tool to validate the microarray’s results. In our study, we found that significant changes occurred during the first stages of *A. flavus* conidia germination.

For GO class analysis, metabolic processes in biological process contain the largest number of DEGs between dormant conidia and conidia 5 h after inoculation. With the exception of the global and overview maps of KEGG enrichment analysis, the DEGs enriched in the nutrient metabolism pathway were the most considerable amount. From a metabolic perspective, the germination process involves a transitioning from a relatively quiescent, dormant state to a germinated state. There needs to be resumption and an increase in metabolic activities including respiration, DNA synthesis, mitosis, cell wall synthesis, RNA and protein biosynthesis throughout germination.

Protein synthesis is vital for germination in *A. fumigatus* and *A. niger* because the protein synthesis inhibitor cycloheximide prevents germ tube formation at moderate concentrations [13,15]. Both protein synthesis and polysome assembly are early events in germination and transcriptome research, with *A. niger* also supporting this conclusion [5]. In our study, pathway enrichment analyses revealed that the genes related to the ribosome and ribosome biogenesis significantly changed after conidia germination. In CZ culture medium, sodium nitrate was the only nitrogen source, and several genes involved in the nitrogen’s metabolism were regulated at the onset of germination. For instance, *NR*, *RT* and *NIT-6* were responsible for converting nitrate into ammonia and increased their transcript levels upon germination. Then, L-amino acids were synthesized after a series of biological processes. L-amino acids are the building blocks of new proteins, and the data showed that transcripts encoding transcription factor *CpcA*, which monitors L-amino acid metabolism increased at the initial stages of germination [28], possibly act as signals for replenishing the pool of L-amino acids intracellularly, which involved the same tendency as conidia germination in *A. niger* [24].

For the energy process, the transcript-encoding enzymes of the tricarboxylic acid cycle (TCA), glycolysis/gluconeogenesis and pentose phosphate pathway were found to be highly abundant in the first stage of germinating conidia but were absent in dormant conidia. Fatty acids can act as a catalyst that starts the gluconeogenesis pathway because they can feed into it. The mRNA profile of genes in *Aspergillus* conidia indicates that gluconeogenesis may be significant for spore survival and germination through the use of stored lipids [15]. In our study, the highly abundant fatty acid degradation and metabolism at 5 h of germination also agreed with this conclusion. Furthermore, the fatty acid elongation and biosynthesis were also highly abundant in the swelling stage, which means lipid metabolism is crucial for the breaking of dormancy.

L-amino acids are also possible substrates for gluconeogenesis after germination and the transcriptome suggested that the proteasome is an organelle that could be functional. Additionally, in contrast to *A. niger* conidia germination, there was a lower abundance of transcripts encoding the proteasome in the 5 h germinating conidia compared to the dormant conidia [18].

In translation, the sequence of codons on mRNA directs the synthesis of a polypeptide chain. This process takes place on the ribosome and the movement of tRNA and mRNA through the ribosome is a complicated process that combines high speeds with high accuracy [29]. The ribosome, a large ribonucleoprotein particle, comprises two subunits (large and small) in all species. In our study, most DEGs related to the ribosome (see details in Table 3 and Figure S1) were upregulated between the first two stages, which means translation activity was highly frequent between these changes. Based on our data, the lipid metabolism pathway was a key pathway for the germ-tube stage, sphingolipids, a type of lipid, are major components of fungal plasma membranes and also an inhibition target that prevents polarized growth in *Aspergillus. nidulans* [30]. Alpha-linolenic acid metabolism was the most influenced pathway in the lipid metabolism pathway, whereby alpha-linolenic acid reduced growth and aflatoxin synthesis after several hours [31]. This finding also supported our research, whereby all four DEGs in this pathway were down-regulated in polarized growth stage.

With conidia germination, respiration become more active, and the antioxidant system simultaneously become more effective [13,18,32]. In this regard, conidia germination affected four DEGs involved in the antioxidant process (Figure 8, Table 3). Notably, the *ss-cat* (spore-specific catalase) and *cat2* (bifunctional catalase-peroxidase) genes were up-regulated, while the *m-cat* (mycelial catalase) gene for protein was down-regulated, and the *cat* (catalase) gene was down-regulated and then up-regulated. These changes in gene abundance could result in mitigating oxidative stress during conidia germination. However, the different gene expression changes need to be explored in the future. Numerous studies have shown that the *cat* (catalase) gene plays an important role in fungal development, aflatoxin biosynthesis and virulence [33]; mycelial catalases transiently protect the fungus from external conditions [34]. However, few studies focus on the antioxidant system during conidia germination, and the function of these genes requires further research.

Coumalic acid and geraniol found in the essential oil of fruit and herbs have been suggested to represent a new class of agents to control *A. flavus* and aflatoxin contamination. The two materials have been reported to inhibit the germination of resting spores of some pathogens by interrupting the antioxidant balance system [35,36,37]. Consistent with previous studies, coumalic acid and geraniol exhibited a potent inhibitory effect on *A. flavus* conidia germination and the *ss-cat*, *cat*, and *cat2* genes were up-regulated at 5 h of germination via the induced antioxidant system imbalance (Figure 8). The changes in these genes might help us figure out the mechanism of *A. flavus* conidia germination. Most importantly, redox genes could be a potential target to inhibit *A. flavus* conidia germination.

In conclusion, the present study found that the many changes in the transcriptome were not correlated with distinct morphological changes during germination. In addition, DEGs related to aflatoxin synthesis were found during polarized growth, which means that the transcription process was triggered in an early stage. In general, RNA-Seq was used to uncover transcriptome changes at the conidia germination of *A. flavus*. Translation, amino acid metabolism and carbohydrate metabolism were the most active pathway in breaking conidia germination. Moreover, lipid metabolism, amino acid metabolism and carbohydrate metabolism were the top three pathways during germ-tube growth. Additionally, the antioxidant system plays a crucial role in conidia germination, and *ss-cat*, *cat* and *cat2* are essential redox genes. However, the further validation of the exact functions and mechanisms of these key DEGs in conidia germination needs to be further studied and might potentially be beneficial in preventing aflatoxin contamination.

## 4. Materials and Methods

### 4.1. Culturing Conditions and Sampling

*Aspergillus flavus* NRRL 3357 was obtained from Prof. Zhumei He (Sun Yat-Sen University, Guangzhou, China) [38]. The strain was grown on Potato Dextrose Agar (PDA) for 7 days at 30 °C to develop mature spores. Spores were then harvested with sterile 0.05% (*w*/*v*) Tween 80 solution. The spore suspension was filtered through 3 sterile layers of lens paper and kept on ice until further processing on the same day, and the spore population was quantified using a hemocytometer. For spore germination, 20 mL of 10^6^ mL^−1^ spores was inoculated in 200 mL liquid Czapek–Dox (CZ) Medium. Three replicates were shaken at 150 rpm at 30 °C for each RNA isolation. At each time point, samples were pooled and centrifuged at 5 °C for 10 min at 3000× *g*. The pellet was frozen in liquid nitrogen for later RNA isolation. Coumalic acid and geraniol were dissolved in ethanol into a 100 mg/mL stock solution, protected from light, and stored at 4 °C. The final concentration of the coumalic acid treatment groups was 200 mg/L and geraniol was 100 mg/L.

### 4.2. Microscopy

For scanning electron microscopy (SEM) analysis, 1 × 10^6^ conidia of *A. flavus* were harvested by centrifugation at 3000× *g* and washed with PBS (phosphate buffered saline, pH 7.4) twice. Then, conidia were fixed in 2.5% glutaraldehyde in PBS for 2 h at room temperature. The conidia were washed with PBS for 3 times, 15 min each, and then the conidia were post-fixed in 1% osmium tetroxide for 1–2 h at room temperature. After that, conidia were washed in PBS for 3 times. The dehydration of samples was achieved by transferring by increasing the concentration of (30–100%) ethanol solutions, and the samples were dried with Critical Point Dryer [39]. The samples were then attached to metallic stubs using stickers and sputter-coated with gold for 30 s. The observations were made on a HITACHI Regulus 8100 SEM (Tokyo, Japan).

### 4.3. Flow Cytometry of Spores

Flow cytometry was used to measure the size of spores (1 × 10^5^) over the first few hours of germination when the conidia were swelling. Liquid CZ medium was inoculated with *A. flavus* conidia at a concentration of 10^6^/mL and shaken at 150 rpm at 28 °C. The samples were collected 5 and 10 h after inoculation. The supernatant was removed, and conidia were washed 3 times with 1 mL Tween 80 (0.01% *v*/*v*) and resuspended in 0.5 mL Tween 80. The sample was then analyzed using flow cytometry (Beckman-CytoFLEX Coulter, Brea, CA, USA). FlowJo software was used to determine the forward scatter (FSC) parameter for each sample, which is a measure of conidial size [25]. The same number of dormant conidia was analyzed as well.

### 4.4. RNA Extraction and RNA-Seq

Total RNA was extracted from conidia using a TRIzol and chloroform RNA extraction protocol, as previously described [5]. Three replicate RNA-Seq libraries were prepared from dormant conidia at 5 h and 10 h after the inoculation of *A. flavus*. A total of the nine libraries were sequenced separately using BGISEQ-500 sequencer. Raw sequencing reads were cleaned by removing adaptor sequences, reads containing ply-N sequences and low-quality reads. Approximately 24,006,405 clean reads were mapped to the Nipponbare reference genome using HISAT [40]/Bowtie [41] tools. After data were mapped, normalization was performed and then FPKM (fragments per kilobase per million mapped reads) was calculated using RESM software [42]. As previously described [43], a false discovery rate (FDR) < 0.01 and absolute value of log2 ratio ≥ 1 were used to identify differentially expressed genes in dormant conidia versus 5 h and 5 h versus 10 h samples.

### 4.5. Real-Time Quantitative PCR

Total RNA from 6 individual *A. Flavus* spore samples in each treatment (0 h, 4 h, 8 h and 12 h) and 2 essential oil supplement groups (after 8 h inoculation) were isolated, and the quality and quantity of RNA were analyzed by using Thermo NanoDrop (Thermo, Waltham, MA, USA). To estimate the accuracy of transcriptome results and for further investigation, 4 DEGs, *ss-cat* (spore-specific catalase), *cat2* (bifunctional catalase-peroxidase), *m-cat* (mycelial catalase) and *cat* (catalase), were selected using Real-time quantitative PCR (RT-qPCR). RT-qPCR was conducted on a Bio-Rad CFX384 Real-Time PCR System (Bio-Rad, Hercules, CA, USA) with TB Green^®^ Premix Ex Taq™ II (Tli RNaseH Plus) (Takara, Dalian, China). The relative amounts of mRNAs were normalized with the housekeeping gene *GAPDH* and were analyzed by the 2^−ΔΔCt^ method.

### 4.6. Statistical Analysis

For spore germination rates and transcriptomic data, statistical analyses were performed using Graphpad Prism (San Diego, CA, USA) for Windows (version 8.00). The data were expressed as mean ± SEM (standard error of mean). Differential effects were analyzed by one-way analysis of variance (ANOVA). A *p* value < 0.05 was considered significant (*), and *p* value < 0.001 was considered extremely significant (***). 

### 4.7. Data Submission

All the amplicon sequencing datasets in this study were submitted to NCBI Sequence Read Archive (SRA) under accession number PRJNA698788.

## Figures and Tables

**Table 1:** Statistical summary of the different conidia RNA-Seq datasets.

Sample	Raw Data Size (bp)	Raw Reads Number	Clean Data Size (bp)	Clean Reads Number	Clean Data Rate ^1^ (%)
0 h	1,206,821,150	24,136,423	1,206,488,600	24,129,772	99.97
0 h	1,206,804,050	24,136,081	1,206,255,800	24,125,116	99.95
0 h	1,206,810,000	24,136,200	1,206,384,900	24,127,698	99.96
5 h	1,206,793,700	24,135,874	1,206,071,500	24,121,430	99.94
5 h	1,206,826,500	24,136,530	1,205,452,250	24,109,045	99.88
5 h	1,206,836,250	24,136,725	1,206,384,850	24,127,697	99.96
10 h	1,206,827,750	24,136,555	1,206,450,400	24,129,008	99.96
10 h	1,206,830,400	24,136,608	1,206,429,400	24,128,588	99.96
10 h	1,206,829,750	24,136,595	1,206,415,650	24,128,313	99.96

^1^ Clean Data Rate (%) = Clean Reads Number/Raw Reads Number.

**Table 2:** The differentially expressed genes grouped by GO, KEGG and enriched pathways of interest between 5 and 10 h.

Pathway	Gene ID	log2Ratio ^a^	Up/Down	Probability	Description
Alpha-Linolenic acid metabolism	7917785	−1.02	Down	0.80373	3-ketoacyl-coA thiolase peroxisomal A precursor, mRNA
	7915336	−1.69	Down	0.80566	FMN binding oxidoreductase, putative, mRNA
	7912988	−1.95	Down	0.86628	NADH-dependent flavin oxidoreductase, putative, mRNA
	7912986	−2.05	Down	0.89858	Fatty-acyl coenzyme A oxidase (Pox1), putative, mRNA
	7910815	2.87	Up	0.929795	40S ribosomal protein S22, partial mRNA
Aflatoxin biosynthesis	7909985	−1.08	Down	0.810561786	PKS-like enzyme, putative, mRNA
	7910374	3.32	Up	0.863160112	short chain type dehydrogenase, putative, mRNA
	7912783	1.45	Up	0.842033907	toxin biosynthesis ketoreductase, putative, mRNA
	7914380	7.38	Up	0.83854578	benzoate 4-monooxygenase cytochrome P450, mRNA
	7911412	5.07	Up	0.865160849	cytochrome P450, putative, mRNA
	7911415	6.16	Up	0.941794345	short-chain dehydrogenase, putative, mRNA
	7911112	3.25	Up	0.826041173	cytochrome P450, putative, mRNA
	7915318	5.90	Up	0.834952351	O-methyltransferase family protein, mRNA
	7912683	8.03	Up	0.80137419	O-methyltransferase, putative, mRNA
	7915318	5.90	Up	0.834952351	O-methyltransferase family protein, mRNA
	7911961	1.56	Up	0.801874375	O-methyltransferase family protein, mRNA

^a^ log2Ratio was determined as the log2 mean value of mRNA abundance at 5 h vs. 10 h.

**Table 3:** The differentially expressed genes grouped by GO, KEGG and enriched pathways of interest between 0 and 5 h.

Pathway	Gene ID	log2Ratio ^a^	Up/Down	Probability	Description
Ribosome	7913639	3.02	Up	0.905636	ribosomal protein YmL41, partial mRNA
	7910217	2.97	Up	0.929728	60S ribosomal protein L31, partial mRNA
	7921558	2.87	Up	0.929755	60S ribosomal protein L1, partial mRNA
	7913089	2.87	Up	0.929914	40S ribosomal protein S20, partial mRNA
	7910815	2.87	Up	0.929795	40S ribosomal protein S22, partial mRNA
Redox genes	7912906	7.00	Up	0.974886	catalase Cat, mRNA
	7920700	−1.68	Down	0.904521	spore-specific catalase CatA, mRNA
	7917068	6.11	Up	0.950608	mycelial catalase Cat1, mRNA
	7918464	1.84	Up	0.850788	bifunctional catalase-peroxidase Cat2, mRNA

^a^ log2Ratio was determined as the log2 mean value of mRNA abundance of 0 h vs. 5 h.

## Data Availability

The data presented in this study are available in this article and supplementary materials.

## Supplementary Materials

The following supporting information can be downloaded at https://www.mdpi.com/article/10.3390/toxins14080560/s1, Figure S1: DEGs in ribosome pathway in *A. flavus* between 0/5 h. Figure S2: DEGs in alpha-linolenic acid metabolism pathway in *A. flavus* between 5/10 h. Figure S3: DEGs in aflatoxin biosynthesis pathway in *A. flavus* between 5/10 h. Excel S1: All DEGs between 0 h vs. 5 h. Excel S2: All DEGs between 5 h vs. 10 h.
