# Supplementary material for: Transcriptional Stages of Conidia Germination and Associated Genes in Aspergillus flavus: An Essential Role for Redox Genes
Source: Toxins (Basel). 2022 Aug 18;14(8):560. doi: 10.3390/toxins14080560 (PMC9412981; doi:10.3390/toxins14080560)
Supplement: Supplementary file 1 [file toxins-14-00560-s001.zip › toxins-1826145--supplementary.pdf]

Supplementary Materials: Transcriptional Stages of Conidia Germination and Associated Genes in Aspergillus Flavus: An Essential Role for Redox Genes

Chong Li, Sifan Jia, Shahid Ali Rajput, Desheng Qi and Shuai Wang

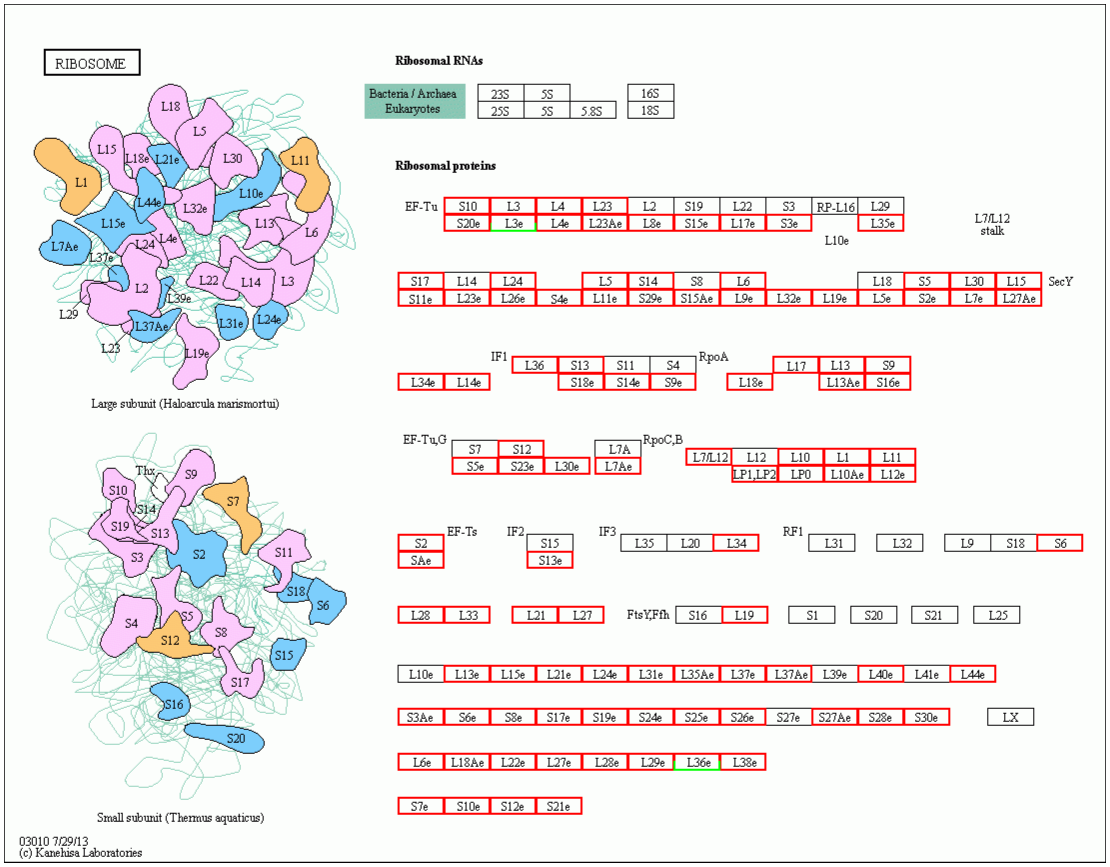

Figure S1. DEGs in ribosome pathway in *A. flavus* between 0/5 h. Square represents different proteins, red represents up-regulated DEGs and green represents down-regulated DEGs.

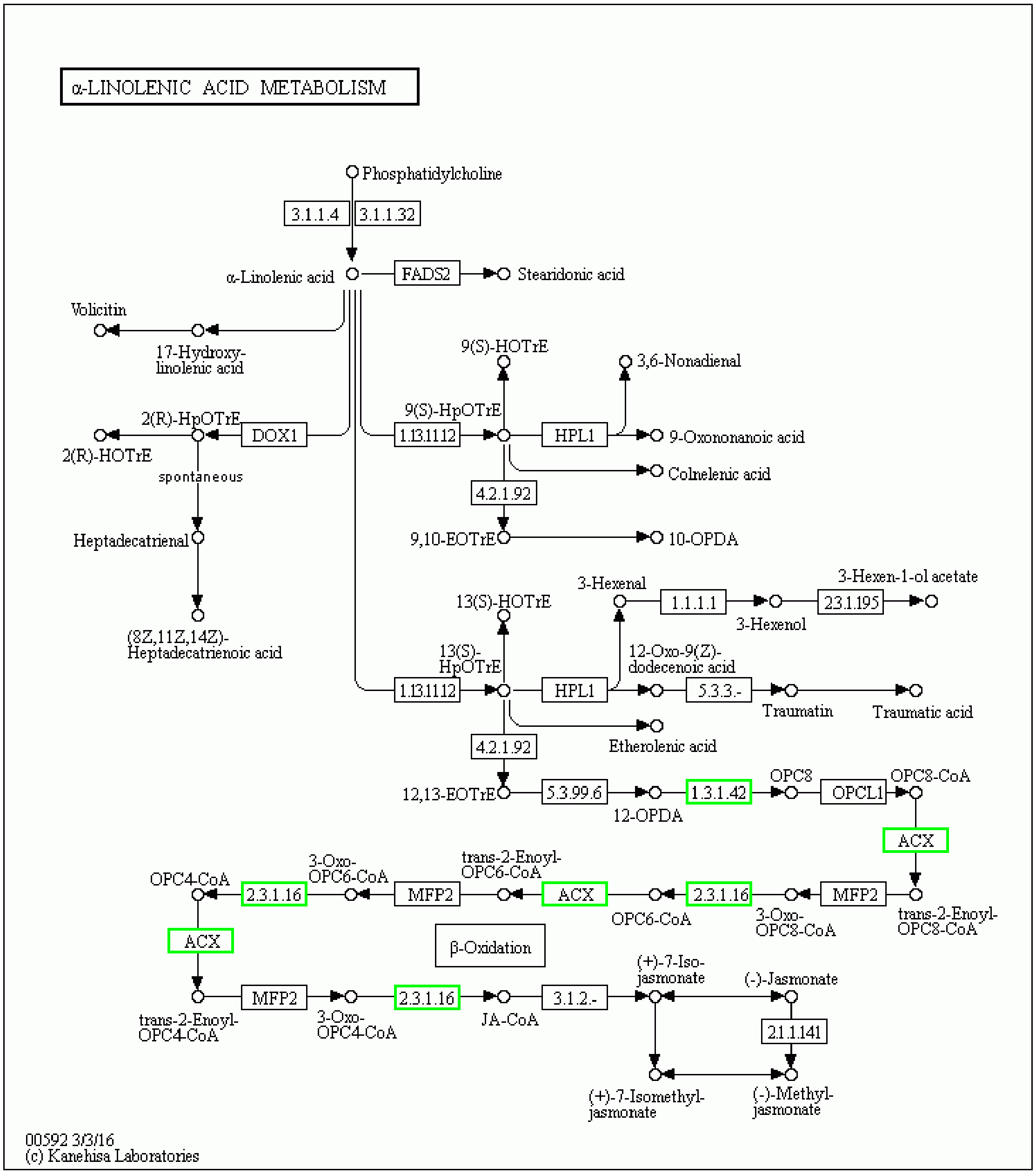

Figure S2. DEGs in alpha-Linolenic acid metabolism pathway in *A. flavus* between 5/10 h. Square represents different proteins, green represents down-regulated DEGs.

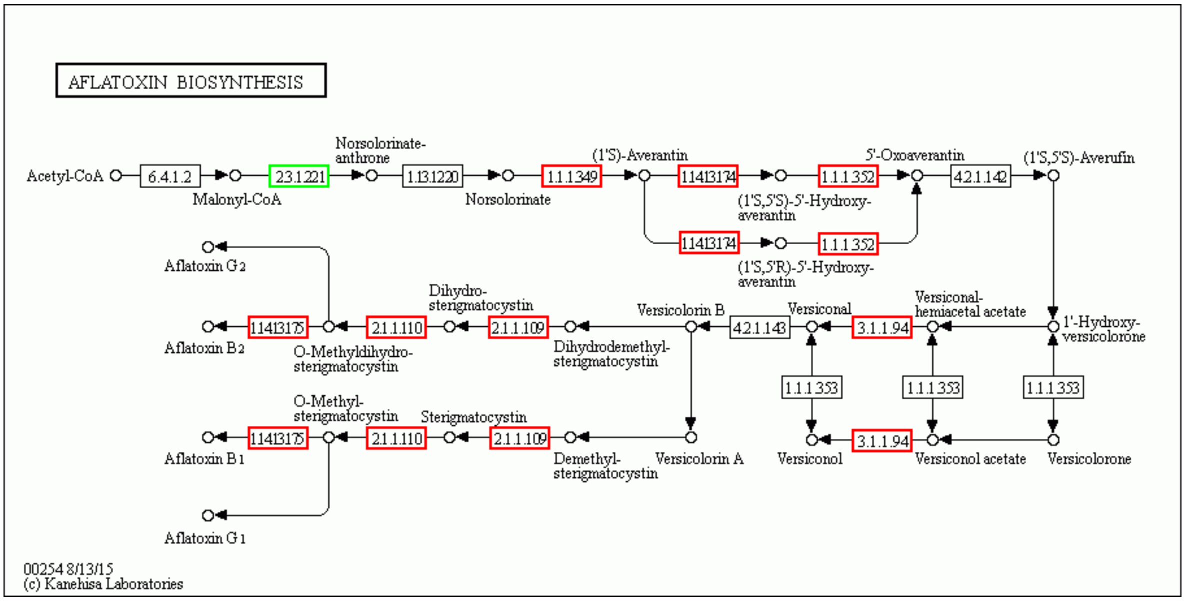

Figure S3. DEGs in aflatoxin biosynthesis pathway in *A. flavus* between 5/10 h. Square represents different proteins, red represents up-regulated DEGs and green represents down-regulated DEGs.
